# Standardized disease-related measures in diabetes research: results from a global consensus process

**DOI:** 10.3389/fpubh.2025.1580416

**Published:** 2025-07-28

**Authors:** Meena Daivadanam, Kristi Sidney Annerstedt, Rajesh Vedanthan, Louise Maple-Brown, Gary Parker, Maia Ingram, Gina Agarwal, Josefien van Olmen, Renae Kirkham, Kirsten Bobrow, Francisco Gonzalez-Salazar, Fanny Monnet, GACD Diabetes Data Standardization Working Group, Aravinda Berggreen-Clausen, Christina Mavrogianni, David Guwatudde, Deksha Kapoor, Edward Fottrell, Elsa Cornejo, Jeroen De Man, Maria Lazo-Porras, Ninha Silva, Puhong Zhang, Violeta Iotova, Xuanchen Tao

**Affiliations:** ^1^Global Health and Migration Unit, Department of Women’s and Children’s Health, Uppsala University, Uppsala, Sweden; ^2^Department of Global Public Health, Karolinska Institutet, Stockholm, Sweden; ^3^Department of Population Health, NYU Grossman School of Medicine, New York, NY, United States; ^4^Menzies School of Health Research, Charles Darwin University, Darwin, NT, Australia; ^5^Department of Endocrinology, Royal Darwin Hospital, Darwin, NT, Australia; ^6^Global Alliance for Chronic Diseases, London, United Kingdom; ^7^Mel and Enid Zuckerman College of Public Health, University of Arizona, Tucson, AZ, United States; ^8^Department of Family Medicine, McMaster University, Hamilton, ON, Canada; ^9^Primary and Interdisciplinary Care Antwerp (ELIZA), Department of Family Medicine and Population Health, University of Antwerp, Antwerp, Belgium; ^10^Department of Family Medicine (DFM) & Centre for Research in Health System Performance (CRiHSP), Yong Loo Lin School of Medicine, National University of Singapore, Singapore, Singapore; ^11^Department of Medicine, Faculty of Health Sciences, University of Cape Town, Cape Town, South Africa; ^12^Division of Health Sciences, University of Monterrey, Monterrey, Mexico; ^13^Center for Population, Family and Health, Department of Sociology, University of Antwerp, Antwerp, Belgium

**Keywords:** type 2 diabetes, cross-contextual research, disease-related measures, standardization, consensus, implementation research

## Abstract

**Background:**

A lack of disease-related consensus measures for type 2 diabetes interventions is a barrier to comparing interventions across various contexts, as well as to implementation and scale-up. This study aimed to use an expert consensus approach to select disease-related measures for type 2 diabetes to facilitate cross-contextual research, as well as the implementation and scaling-up of initiatives.

**Methods:**

The study was conducted using a two-phased cross-sectional design consisting of an online survey among research experts in 17 diabetes projects working in a global context, followed by an online modified Delphi panel comprised of reviewers with domain-specific expertise from different income settings who were not survey participants.

**Results:**

Out of 153 measures from 11 domains assessed, 49 were classified as core, 58 as optional, and 46 were excluded. The domains and measures spanned several categories, including demographics, medical history, medication adherence, health behaviors, anthropometric measures, biochemical measures, and quality-of-life-related issues.

**Conclusion:**

The core dataset of selected measures in type 2 diabetes may provide a standardized approach for determining which data should be collected. This can facilitate transnational comparisons between or within implementation projects to advance global diabetes research.

## Introduction

The global burden of type 2 diabetes continues to escalate at alarming rates, with recent estimates of approximately 463 million (1 in 11) adults living with type 2 diabetes, and with an estimated cost of USD 727 billion worldwide (1). Diabetes has drastic implications on morbidity and mortality, as well as economic and social well-being, and these repercussions disproportionately impact people living in low- and middle-income countries (LMICs) and underserved populations in high-income countries (HICs) (1). The implementation of proven evidence-based interventions is lagging, and these efforts often do not reach the people who need it the most (2). This highlights the need to reconsider methods, indicators, and strategies from an implementation perspective that considers ‘real-life’ conditions as opposed to more controlled conditions and across different contexts (1, 3).

There is a lack of standardized disease-related consensus measures for type 2 diabetes interventions across different contexts, which poses a significant barrier to the implementation and scale-up of these interventions. Currently, existing global and national diabetes related guidelines, such as the Guide for Diabetes Epidemiology Studies from the International Diabetes Federation (IDF) (4), the Standards of Medical Care in Diabetes from the American Diabetes Association (5) or the diabetes management guidelines from the United Kingdom (6), are not comparable across projects. This hampers global progress.

Implementation research provides an overall approach for concerted and coordinated evaluation of implementation efforts across multiple contexts, focusing on three outcomes: implementation outcomes, service outcomes, and client outcomes. While implementation outcomes (e.g., acceptability, feasibility, fidelity, adoption, and sustainability) are disease-agnostic, service outcomes (e.g., efficacy, safety, and effectiveness) and client outcomes (e.g., satisfaction, functional improvement, and overall well-being) usually have specificities related to the condition at stake (7–9). As such, it is essential to reach consensus on harmonizing data definitions, measures, and core outcomes to compare and contrast diabetes-specific intervention outcomes (i.e., service outcomes and client outcomes) on a transnational level.

In this study, we described an expert consensus process to select disease-related measures that may be considered ‘core’ (i.e., the minimal number of measures recommended for data collection) for research projects related to type 2 diabetes. This process, and the adoption of its results, will facilitate cross-setting learning on implementation and outcomes (10), thereby allowing the global research community to identify best practices for wider implementation and scale-up.

## Methods

### Study setting: Global Alliance for Chronic Diseases (GACD)

The GACD was established in 2009 and includes 12 national or regional funding agencies across the globe (11). The GACD aims to address the high burden of chronic diseases in LMICs and among underserved populations in HICs experiencing health disparities by coordinating joint research funding calls across all participating funding agencies. All projects funded under the GACD have an implementation science focus. The Data Standardization Working Group, a subgroup of 17 diabetes research projects (2014–2019), recognized the need to harmonize measures across diverse contexts in diabetes research. It is in this context that we pursued the current project.

### Study design

This study was conducted using a two-phase cross-sectional design. In phase I, the Data Standardization Working Group conducted an online survey among the GACD researchers within the participating 17 diabetes projects (12). The aim was to identify and reach consensus on disease-related measures to be collected in the diabetes-related projects. Results of the survey informed the development of phase II, which employed an online modified Delphi panel (ODP) (13) comprising reviewers with domain-specific expertise from different income settings who were not part of the phase I survey or the Data Standardization Working Group (14). The Delphi technique is often used to systematically combine expert opinion to arrive at an informed group consensus on a complex problem (15–17). The objective of the ODP panel survey was to resolve conflicting information from phase I and confirm the final recommended set of consensus measures.

### Phase I

#### Participants

The principal investigator of each project in the GACD diabetes program (*n* = 17) was contacted and asked to nominate 1–3 researchers from their team with domain expert knowledge who could be included as participants and fill out the survey.

#### Data collection

The Working Group used the GACD data dictionary that was compiled by the first cohort of GACD implementation projects (18) to identify a broad set of potential measures for the online survey. The survey was based on 11 domains (Supplementary Table S2): demographics, anthropometry, behavioral measures, bio-chemical measures, dietary measures, health care utilization, medical history, medication and adherence, physical activity, quality of life, stress, and support systems. The survey questions determined whether each measure was collected in the respective diabetes projects and the definition used. When projects did not use a particular measure, they were asked if they would consider adding it. The phase I survey tool was piloted among five projects and modified following each pilot session.

#### Data analysis

**Figure 1:**
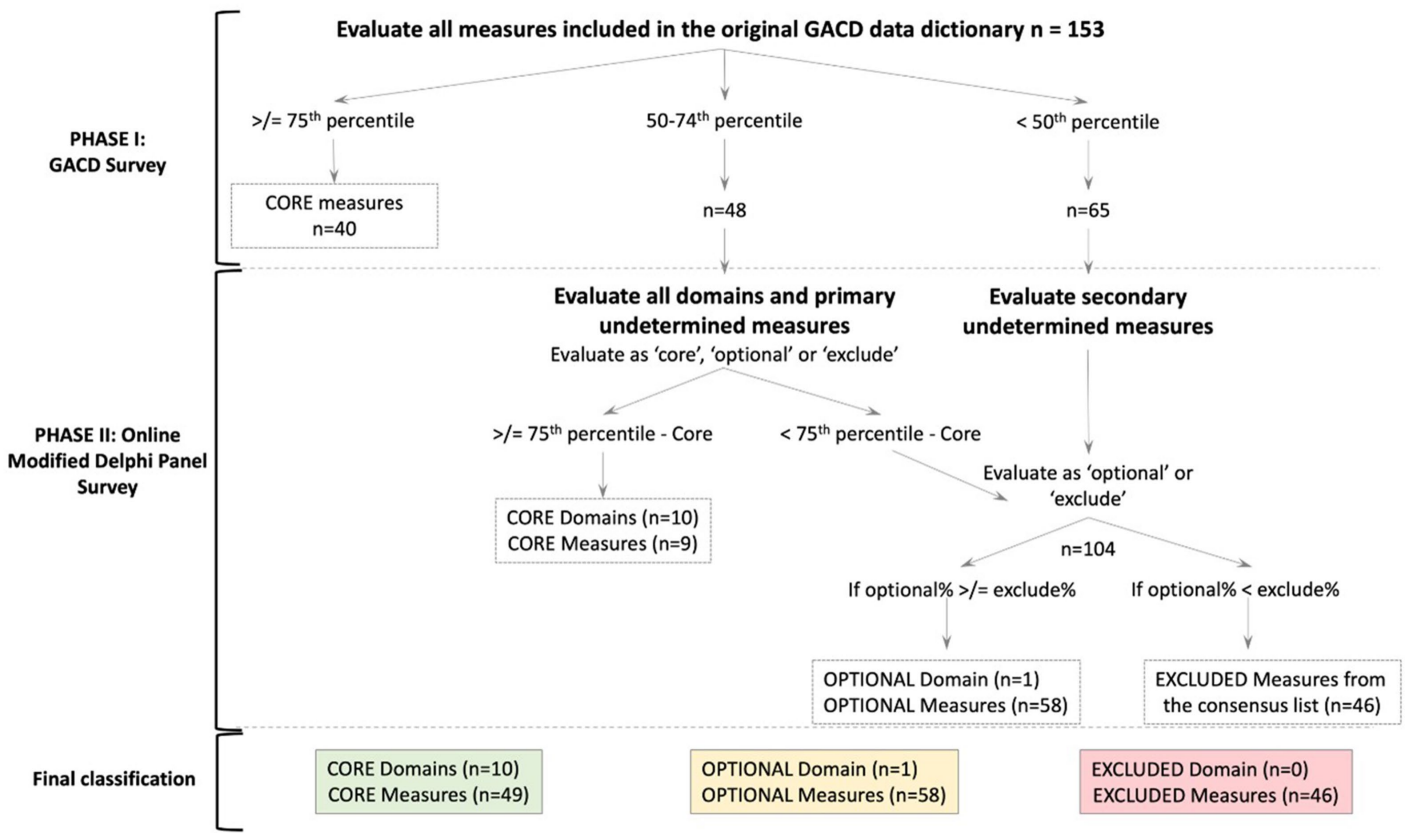
Consensus decision algorithm. The number of domains or measures at each cut-off or level is denoted in parentheses as (*n*=). The dotted line box indicates the decision at each phase. The final classification is in solid color-coded boxes.

Descriptive statistics were used to determine the most common measures across the 17 diabetes projects. The consensus decision algorithm (Figure 1) depicts the process followed. The 75th and 50th percentiles of projects collecting data in a domain were ascertained and used as the two cut-offs to categorize each of the measures as core or undetermined, respectively, within the 11 domains. These cut-offs were based on the interquartile distribution of responses, rather than a simple majority, to better reflect their relative frequency across diverse settings. Measures reported by projects at or above the 75th percentile were classified as core and automatically included in the final set, as this indicates widespread use and essential relevance.

The ODP panel was consulted on two sets of variables: those between the 50th and 75th percentile (classified as *“primary undetermined”*) and those below the 50th percentile (classified as *“secondary undetermined”*). If a parent question had a response rate in a specific cut-off range in the phase I survey, the child question was treated as belonging to the same response category, irrespective of the actual response. An example of a parent–child question is as follows: “Are you on oral hypoglycaemic agents (parent)? If yes, what medicine and dose (child)?”. The domain ‘support systems’ was an exception in this classification system as it was only collected by three projects in the phase I survey. Following discussions in the Working Group, it was decided to include all the measures in this domain in the primary undetermined group for further analysis.

### Phase II

#### Participants

The diversity of the expert panel across disciplines, geography, and areas of expertise was considered crucial in identifying measures that address the different aspects of prevention and management of diabetes. Based on these criteria, Working Group members nominated 88 experts to represent a well-balanced international panel based on their professional expertise and academic experience in one or more specific fields relevant to implementation research. All 88 experts were invited to participate in the OPD via email from July to September 2018. Three automatic reminders were sent during this period.

#### Data collection

The ODP panel survey used in phase II was developed based on the results of the phase I survey. Since phase II was designed primarily to resolve conflicting consensus from the phase I survey, only measures with a response rate less than the 75^th^ percentile (excluding measures related to ID of devices) were included. The experts were first asked their expert opinion on the domains (not individual measures) as defined in the instructions as ‘core’ i.e. all implementation research projects should include in their data collection, ‘optional’ i.e. depending on the research question, the variable could be included and finally ‘excluded’ i.e. the variable should not be collected. All measures classified as *primary undetermined* were listed, and the ODP experts were requested to classify them as ‘core’, ‘optional,’ or ‘excluded’. The experts were also asked to confirm the classification of measures from the *secondary undetermined* group as ‘optional’ or ‘excluded’ and name any additional measures they considered relevant but were not included in either phase I or II surveys. The software Research Electronic Data Capture (REDCap) was used to create and facilitate the ODP survey (19). The phase II survey was piloted among three members of the GACD research network, and modifications were made based on the responses.

#### Data analysis

Descriptive statistics (i.e., mean, median, interquartile range) for each variable were calculated by the 11 domains (Supplementary Table S1). The domains and measures were ultimately classified as ‘core’, ‘optional’, and ‘excluded’ accordingly. The final consensus measure list was reached using a consensus decision algorithm, as shown in Figure 1. We used the Recommendations for Conducting and REporting of DElphi Studies (CREDES) to guide the reporting of our findings (20).

### Ethical considerations

As this was an expert consensus study, no ethical approval was needed as we did not use individual-level identifiable data. The research topic was deemed non-sensitive, and the Delphi panel members are domain experts who consented to be involved in the study professionally (21). All experts were also asked if they would like to be included in the acknowledgement section of the manuscript, and for those who agreed, their names are included under acknowledgements.

## Results

In phase I, 16 people filled out the form from 13 projects (76% response rate) between March 2017 and April 2018. In phase II, 32 experts participated in the ODP survey (response rate of 36%), and their area of expertise is presented in Table 1. Experts could select more than one area of expertise. Six experts were from low-income, 13 from middle-income, and 13 from high-income countries, with a response rate of 35, 72, and 65%, respectively. Experts from LMICs were represented in all areas of expertise. Across all areas, the median years of experience were more than 10 years.

**Table 1:** Area of expertise among members of the online Delphi panel survey, *n* = 32.

Area of expertise*	Median years of experience (IQR)	Participation in the survey			
		LICs	MICs	HICs	Total
		*n* = 6	*n* = 13	*n* = 13	*n* (%)**
Diabetes	17 (11–20)	33%	62%	46%	16 (50)
Endocrinology	20 (17–30)	17%	23%	8%	5 (16)
Cardiometabolic risk	17 (12–20)	0%	38%	38%	10 (31)
Primary care	13 (3–25)	17%	15%	31%	7 (22)
Behavioral science	15 (8–25)	0%	31%	15%	6 (19)
Epidemiology	11 (8–20)	33%	85%	46%	19 (59)
Cardiovascular disease (CVD)	14 (10–15)	83%	38%	23%	13 (41)
Health systems	14 (10–16)	33%	38%	46%	13 (41)
Program evaluation	10 (10–15)	17%	8%	23%	5 (16)
Health economics	16 (11–25)	17%	0%	23%	4 (13)
Pregnancy (obstetrics)	16 (12–20)	0%	8%	15%	3 (9)
Nutrition	15 (6–25)	0%	23%	31%	7 (22)
Physical activity	20 (11–20)	0%	23%	31%	7 (22)
Pediatrician	14 (10–18)	0%	15%	0%	2 (6)
Social science	20 (12–28)	0%	8%	23%	4 (13)

IQR, Interquartile range; LIC, low-income countries; MIC, middle-income countries; HIC, high-income countries; *Area of expertise was not mutually exclusive; **Percentage out of total respondents (*n* = 32).

We used the consensus decision algorithm shown in Figure 1 to create the final consensus measure list. Ten of the 11 domains were deemed core, and one was deemed optional (support system). A total of 153 measures were assessed in phase I by the GACD survey, which identified 40 measures as core. Among the remaining 113 measures, 48 were classified between the 50th and 75th percentile, and 65 below the 50th percentile. These two categories were assessed by the ODP in phase II. The domains of each set of measures (*n* = 11) were also assessed simultaneously. Nine of the 48 between the 50th and 75th percentiles were ultimately classified as core, making a total of 49 core measures.

Table 2 outlines the domains, recommended core measures, and the phase to determine their recommendations. For example, in domain 1, the participant’s unique identifier was recommended as a core measure from the GACD survey, while participant age in years was recommended to be core through the Online Delphi Panel (ODP) survey. The measures in the domains are described as core, optional, and excluded. Supplementary Table S2 provides the classification of all measures by domain. The expert recommendations from the ODP survey (phase II) did not significantly differ between experts from LMICs compared to HICs for any of the 113 measures.

**Table 2:** Recommendation of core measures by domain* based on Phase I and II analyses.

Recommended domains and core measures	Phase I: GACD Survey	Phase II: Online Delphi Panel (ODP) Survey			
	GACD projects assessing *n* (%)	# Domains assessed by experts *n* (%)	Core measures assessed by experts *n* (%)	Optional measures assessed by experts *n* (%)	Excluded measures assessed by experts *n* (%)
DOMAIN 1: Demographics	**13/13 (100)**	**27/32 (84)**	**25/27 (93)**		
Participant’s unique identifier number	13 (100)		-	-	-
Participant age in years	11 (85)		22 (81)	5 (19)	
Participant sex	12 (92)		-	-	-
Highest participant education	12 (92)		-	-	-
DOMAIN 2: Anthropometry	**12/13 (92)**	**23/32 (72)**	**22/23 (96)**		
Systolic blood pressure	12 (100)		-	-	-
Diastolic blood pressure	12 (100)		-	-	-
Participant height	12 (100)		-	-	-
Participant waist circumstance	11 (92)		-	-	-
Participant weight	12 (100)		-	-	-
DOMAIN 3: Behavioral measures	**12/13 (92)**	**25/32 (78)**	**25/25 (100)**		
Currently smoke cigarettes	12 (100)		-	-	-
Currently use smokeless tobacco/chewing tobacco/snuff	7 (58)		20 (80)	4 (16)	1 (4)
Frequency of tobacco (smoking or smokeless) use	10 (83)		-	-	-
Alcohol frequency over the past 12 months	9 (75)		-	-	-
Blood sugar healthcare professional^†^	4 (33)		22 (88)	2 (8)	1 (4)
DOMAIN 4: Biochemical measures	**12/13 (92)**	**23/32 (72)**	**19/23 (83)**		
Fasting blood glucose measured in the past 12 h	5 (42)		-	-	-
Unit for fasting blood glucose measurement	7 (58)		-	-	-
Baseline time for blood glucose concentration^‡^	4 (33)		20 (90)	1 (5)	1 (5)
Unit of baseline glucose concentration	5 (42)		-	-	-
Glycated hemoglobin concentration	9 (75)		-	-	-
Total cholesterol	5 (42)		-	-	-
High-density lipoprotein	5 (42)		-	-	-
Cholesterol: High-density lipoprotein ratio	5 (42)		-	-	-
DOMAIN 5: Dietary measures	**10/13 (77)**	**21/32 (66)**	**20/21 (95)**		
Frequency of fruit consumption in a week	9 (90)		-	-	-
Frequency of fruit servings on a day when fruit is consumed	7 (70)		-	-	-
Frequency of vegetable consumption in a week	9 (90)		-	-	-
Frequency of vegetable servings on a day when vegetables are consumed	7 (70)		-	-	-
DOMAIN 6: Health care utilization	**10/13 (77)**	**27/32 (84)**	**22/27 (81)**		
Medical advice in the last 3 months	5 (50)		-	-	-
Frequency of medical advice in the last 3 months	4 (40)		-	-	-
Admitted to the hospital in the last year	4 (40)		-	-	-
Health insurance	5 (50)		-	-	-
Type of health insurance	5 (50)		-	-	-
Payment of health insurance	4 (40)		-	-	-
DOMAIN 7: Medical history	**13/13 (100)**	**24/32 (75)**	**22/24 (92)**		
History of hypertension	12 (92)		-	-	-
Hypertension medication	11 (85)		-	-	-
History of CVD: doctor informed	8 (62)		20 (83)	3 (13)	1 (4)
History of stroke: doctor informed	7 (54)		21 (88)	3 (12)	
History of diabetes: doctor informed	11 (85)		-	-	-
Diabetes medication	12 (92)		-	-	-
Insulin use	8 (62)		20 (83)	4 (17)	
History of chronic kidney disease: doctor informed	6 (46)		20 (83)	4 (17)	
DOMAIN 8: Medication and adherence	**7/7 (100)**	**24/32 (75)**	**19/24 (79)**		
Forget to take medication 1	5 (71)		-	-	-
Adherence to medication when feeling good^†^	4 (57)		-	-	-
Clinical data collection month	4 (57)		-	-	-
Clinical data collection year	4 (57)		-	-	-
DOMAIN 9: Physical activity	**12/12 (100)**	**23/32 (72)**	**20/23 (87)**		
Physical activity time^‡^	10 (83)		22 (100)	-	
DOMAIN 10: Quality of life and stress	**8/8 (100)**	**21/32 (66)**	**19/21 (90)**		
General perception	6 (75)		-	-	-
Health assessment	7 (88)		-	-	-
Nervous^†^	4 (50)		-	-	-
Worry^†^	3 (38)		-	-	-
Difficulties	4 (50)		-	-	-

*Domains are based on the 75th percentile cut-off in Phase I, and further details are available in Supplementary Tables S1, S2. ^†^ Denotes the variable is part of a validated scale. ^‡^ 22 of the 23 experts replied. In Phase I (GACD Survey), the *n* refers to the number of projects collecting that measure. In Phase II, the *n* refers to the number of experts who assessed that domain and/or measured it by the denominator.

### Demographics

Out of 10 measures in the demographic domain, the recommendations were four cores, five optional, and one excluded, based on the ODP recommendation.

Participants’ unique identifier, age, sex, and education were finalized as the core demographic measures, whereas income, employment, household size, current employment, and occupation were optional.

### Anthropometry and behavioral measures

Out of 22 measures in these two domains, #10 and #12 were recommended as core and optional, respectively. Blood pressure, height, weight, and waist circumference were identified as core in the phase 1 (GACD) survey, whereas pulse rate and hip circumference were deemed optional by the ODP survey. Concerning behavioral measures related to alcohol and tobacco use as well as blood, foot, and eye examination, only current use and frequency of smoking and smokeless tobacco, along with the frequency of alcohol consumption, were finalized as core. Blood glucose measured by a healthcare professional was included as core and is part of the validated scale, the Summary of Diabetes Self-Care Activities (SDSCA) (22).

### Diet and physical activity

From 19 measures in the dietary and physical activity domains, #5, #10, and #4 were recommended to be core, optional, and excluded, respectively. The frequency of consumption of fruits and vegetables and time spent in physical activity were assessed as core. Other measures related to added salt consumption, other food components, and sedentary behavior were assessed as optional.

### Biochemical measurements

From the 20 measures assessed, #8, #3, and #9 were recommended to be core, optional, and excluded, respectively. Fasting blood glucose and HbA1c (including their units of measurement) as well as total cholesterol, HDL cholesterol, and total to HDL cholesterol ratio were assessed as core. Low-density lipoprotein (LDL), albumin: creatinine ratio, and the unit for 2-h glucose concentration were considered optional.

### Health care utilization

From the 20 measures in this domain, #6, #4, and #10 were recommended to be core, optional, and excluded. Medical advice in the last 3 months, including its frequency, number of hospital admissions in the past year, and measures related to health insurance, were assessed as core. Last routine check-up, treatment provider, payment, and location were included as optional.

### Medical history

Out of 18 measures in this domain, #8, #1, and #9 were recommended to be core, optional, and excluded. History of hypertension, diabetes, stroke, cardiovascular disease (CVD), and chronic kidney disease, insulin use, and medication for hypertension and diabetes were assessed as core, whereas foot ulcer in the past year was assessed as optional.

### Medication and adherence

Out of 14 measures in this domain, #3, #7, and #4 were recommended to be core, optional, and excluded. This domain included measures from a validated scale (MARS) (23). Ever forgetting to take medication and stopping medication when feeling good were assessed as core together with the month and year of the said data collection. Measures related to carelessness in taking medication, side effects, and their effect on medication adherence, injectables, and complementary medicine were assessed as optional.

### Quality of life and stress

From the 20 measures in this domain, #5, #6, and #9 were recommended to be core, optional, and excluded. This domain included measures from a validated scale (GAD7) (24). Measures related to general perceptions and health assessment were deemed core.

### Support systems

Out of 10 measures in this domain, all were assessed as optional.

### Scales validated in specific contexts

The GACD and ODP surveys included measures related to three separate validated scales (MARS, GAD7, and SDSCA) as part of the original GACD data dictionary. In the final Working Group meeting on results, these validated scales were specifically discussed since specific questions within the scales were evaluated at different levels (i.e., core, optional, and excluded). Based on a statistical perspective and the assumptions inherent in the scale and its scoring system, the Working Group recommended that validated scales should ideally not be split. However, if they are, they should be considered unvalidated and separate questions, independent of the original scale. In such a case, the recommendations of the consensus process described in Table 2 should be followed.

## Discussion

To our knowledge, this is the first attempt at drafting consensus health-related measures for disease-related outcomes of type 2 diabetes interventions across global contexts. This global consensus study, involving experts and stakeholders from all country-income categories, yielded 11 core domains and 49 core, 58 optional, and 46 excluded measures, respectively, for health-related measures for implementation and scale-up of type 2 diabetes intervention research studies. The domains and measures span several categories, including demographics, medical history, medication adherence, health behaviors, anthropometric measures, biochemical measures, and quality-of-life-related issues. This research has built upon the foundation of the first iteration of data harmonization efforts by previous GACD researchers (22).

In the absence of disease-related consensus measures relevant for implementation research, projects have relied on a wide range of approaches, such as clinical outcomes at the patient level or proxy indicators (i.e., desired behavior change) to gage the effects of implementation. The consensus on disease-specific measures from our study fill a gap in adapting evidence-based diabetes interventions to practice. These measures should be combined with the more disease-agnostic measures, such as feasibility, acceptability, and adoption. These standardized measures will allow diabetes-focused implementation research to establish and compare the process and outcomes of interventions across a variety of contexts (23).

### Variation in perception of measures

Variation in responses of the survey and ODP resulted in certain measures being deemed optional despite being intrinsic to most HIC research projects. For instance, measures of SES readily collected in HICs, such as income and employment, were classified as less relevant by experts at the global level. This likely reflects challenges related to the sensitivity of these questions, variability in definitions, and limited availability or reliability of such data in some settings. Income, for example, is a complex and context-dependent measure, and collecting accurate information can be difficult in environments where income sources are diverse or culturally sensitive to report. Thus, more discussion is needed to identify measures that capture this concept in different contexts (e.g., living conditions). A vast body of evidence illustrates the difficulties associated with estimating income in LMIC settings (25). Consumption of fruit and vegetables was considered core, while other dietary behaviors, such as overconsumption of sugary drinks, were rated as less important. This may evolve as diets change and over-nutrition dominates in more settings (26).

Quality of life around stress was considered core in the survey, which marks a shift in the focus on and support of patient-reported outcomes in diabetes research (27). It is also considered a cross-disease core indicator used in the context of growing multi-morbidity. With the growing awareness of the connection between depression and anxiety with diabetes, the recognition of stress-related variables in this domain is important.

Issues related to certain domains, like support systems classified as optional, may have varying relevance depending on the aim of a specific project, whereas other core domains may apply to all projects on diabetes. In the bio-chemical measurement domain, the relevance of certain measures differed according to the specific setting, e.g., fasting blood sugar versus HbA1c in terms of cost, availability, setting, accuracy, etc.

### Methodological considerations

The process of consideration in the ODP utilized in this study may have introduced a selection bias. Indeed, the Delphi panel experts included indicators that were excluded in Phase 1 because they did not reach the 75^th^ percentile. The ODP was intended to complement the survey and add a qualitative appreciation, allowing for a more in-depth consideration of the measures and a second opportunity to reflect upon the inclusion of all measures. However, it is possible that the GACD Working Group was more selective in determining the core measures due to pragmatic considerations about the feasibility of data collection for each variable. Overall, we found no significant difference between HIC and LMIC experts in their evaluation of domains and measures.

Another limitation is that some core measures were selected based on frequency of use, which does not necessarily guarantee quality. The measures subject to the Delphi panel method were evaluated based on other criteria (such as expert knowledge and experience). This also meant that the less commonly used measures could never become core measures, which is quite a strong assumption, as the focus of implementation may differ between projects. Furthermore, nominating the Delphi panel experts involved a degree of subjectivity. The selection of Delphi panel experts involved a degree of subjectivity. Although the response rate was modest (36%), the panel ultimately included 32 experts with diverse backgrounds across regions, disciplines, and practice settings. This level of participation is comparable to response rates reported in similar Delphi studies (15, 28). While the composition of the panel supports the credibility of the findings, the possibility of non-response bias cannot be excluded. Future studies may benefit from repeating the process to enhance the robustness of the results. Since the consensus was based on expert opinion, other measures of importance not captured by our process may exist, and some results may not align with existing clinical guidelines or across contexts.

Finally, the GACD data dictionary version 2.2 served as the starting point for this study. As such, some limitations of that version may carry over, e.g., specific questions rather than entire scales were included in the data dictionary, and these were not revisited, such as questions from the validated Jackson Heart Study measures. It was challenging to compare and evaluate entire scales against each other versus the component questions of individual scales. One outcome of the process was that previously validated questionnaires may now be split into component questions, e.g., the MARS adherence questionnaire has two questions that are core (forget to take medication and adherence to medication when feeling good) and two are optional (carelessness to taking medication and side effects influence medication adherence). While this approach allows flexibility and reduces data collection burden, it may compromise the psychometric integrity and comparability of these measures. The implications are not yet investigated and will require comparing findings from studies that use only core questions versus those that use all four.

### Future directions

The consensus process yielded some unexpected results in that certain measures deemed as core in the ODP in phase II of our study were not classified by the experts involved in phase I, for example, SES (income) and individual lipid measures. Further consensus work needs to be done to resolve this. It is also crucial that the process of classification of core measures is flexible and easy to update as new research scales, measures, guidelines, and implementation approaches emerge (i.e., the relationship between sleep and health outcomes). Also, future implementation research data harmonization/consensus efforts focusing on implementation outcomes are needed. We recommend a process similar to the one used in this study, which is consensus-driven and builds upon the efforts of existing implementation science projects in LMIC settings. Moreover, multimorbidity consensus measures are relevant to include in the context of increasing multimorbidity, including the development of cross-disease indicators such as quality of life, as it relates to feelings of control over one’s health.

In conclusion, this dataset was developed through a consensus process, and the results demonstrate that it is possible to develop a core dataset of selected health-related measures for type 2 diabetes research across HIC & LMIC settings. Usage of this dataset may save time in determining what data should be collected and may facilitate transnational comparisons between or within projects to advance research globally. As each project independently included indicators that they felt were important to their research design, this study forms a base upon which future efforts can build to expand and augment the measures to be prioritized for research related to NCDs. Further consensus discussions will be required to update the measures.

## Data Availability

The raw data supporting the conclusions of this article will be made available by the authors, without undue reservation.

## Group members of GACD Diabetes Data Standardization Working Group

Aravinda Berggreen-Clausen, Christina Mavrogianni, David Guwatudde, Deksha Kapoor, Edward Fottrell, Elsa Cornejo, Jeroen De Man, Maria Lazo-Porras, Ninha Silva, Puhong Zhang, Violeta Iotova, and Xuanchen Tao.

## References

[1] International Diabetes Federation. (2021) IDF diabetes atlas. Available online at: http://www.idf.org/about-diabetes/facts-figures. (Accessed May 14, 2021).

[2] Chan JCN Gregg EW Sargent J Horton R. Reducing global diabetes burden by implementing solutions and identifying gaps: a lancet commission. Lancet. (2016) 387:1494–5. doi: 10.1016/S0140-6736(16)30165-9, PMID: 27061676

[3] World Health Organization. A guide to implementation research in the prevention and control of non-communicable diseases. Geneva: World Health Organization (2016).

[4] International Diabetes Federation. (2021) IDF guide for diabetes epidemiology studies. Available online at: https://diabetesatlas.org/idf-guide-for-epidemiology-studies/. (Accessed May 14, 2021)

[5] American Diabetes Association. Standards of medical care in diabetes. Diabetes Care. (2021) 44:S226–32. doi: 10.2337/dc21-S002

[6] National Institute for Health and Care Excellence. (2021) Type 2 diabetes in adults: management NICE guideline. Available online at: www.nice.org.uk/guidance/ng28. (Accessed March 4, 2021).

[7] Proctor EK Bunger AC Lengnick-Hall R Gerke DR Martin JK Phillips RJ. Ten years of implementation outcomes research: a scoping review. Implement Sci. (2023) 18:31. doi: 10.1186/s13012-023-01286-z, PMID: 37491242 PMC10367273

[8] Peters DH Adam T Alonge O Agyepong IA Tran N. Implementation research: what it is and how to do it. BMJ. (2013) 347:f6753. doi: 10.1136/bmj.f6753 24259324

[9] Proctor EK Landsverk J Aarons G Chambers D Glisson C Mittman B. Implementation research in mental health services: an emerging science with conceptual, methodological, and training challenges. Adm Policy Ment Health Ment Health Serv Res. (2009) 36:24–34. doi: 10.1007/s10488-008-0197-4, PMID: 19104929 PMC3808121

[10] Daivadanam M Ingram M Sidney Annerstedt K Parker G Bobrow K Dolovich L. The role of context in implementation research for non-communicable diseases: answering the ‘how-to’ dilemma. PLoS One. (2019) 14:e0214454. doi: 10.1371/journal.pone.0214454, PMID: 30958868 PMC6453477

[11] Global Alliance for Chronic Diseases. (2024). GACD - International funding agencies; the mainstay of the alliance. Available online at: https://www.gacd.org/about/people-and-organisation/associate-members. (Accessed November 22, 2024).

[12] Global Alliance for Chronic Diseases. (2024). Projects. Available online at: https://www.gacd.org/research/projects?research-programmes=diabetes&programme-countries=. (Accessed November 22, 2024).

[13] Taylor E. We agree, don’t we? The Delphi method for health environments research. HERD. (2019) 13:11–23. doi: 10.1177/1937586719887709 31887097

[14] Global Alliance for Chronic Diseases. (2024). GACD working groups: data standardisation. Available online at: https://www.gacd.org/research/research-network/gacd-working-groups. (Accessed November 22, 2024).

[15] Hasson F Keeney S McKenna H. Research guidelines for the Delphi survey technique. J Adv Nurs. (2000) 32:1008–15. doi: 10.1046/j.1365-2648.2000.t01-1-01567.x, PMID: 11095242

[16] Dalkey NC. The delphi method: An experimental study of group opinion. Santa Monica, CA: RAND Corporation (1969).

[17] Linstone H Turoff M. The delphi method: techniques and applications. Massachusetts: Addison-Wesley (1995).

[18] Global Alliance for Chronic Diseases. (2024). GACD researcher-developed resources. Available online at: https://www.gacd.org/resources/researchers-and-students/open-access-resources. (Accessed November 22, 2024).

[19] Harris PA Taylor R Thielke R Payne J Gonzalez N Conde JG. Research electronic data capture (REDCap)—a metadata-driven methodology and workflow process for providing translational research informatics support. J Biomed Inform. (2009) 42:377–81. doi: 10.1016/j.jbi.2008.08.010, PMID: 18929686 PMC2700030

[20] Jünger S Payne SA Brine J Radbruch L Brearley SG. Guidance on conducting and reporting DElphi studies (CREDES) in palliative care: recommendations based on a methodological systematic review. Palliat Med. (2017) 31:684–706. doi: 10.1177/0269216317690685, PMID: 28190381

[21] Reynolds J Crichton N Fisher W Sacks S. Determining the need for ethical review: a three-stage Delphi study. J Med Ethics. (2008) 34:889–94. doi: 10.1136/jme.2008.025056, PMID: 19043116

[22] Toobert DJ Hampson SE Glasgow RE. The summary of diabetes self-care activities measure: results from 7 studies and a revised scale. Diabetes Care. (2000) 23:943–50. doi: 10.2337/diacare.23.7.943, PMID: 10895844

[23] Thompson K Kulkarni J Sergejew AA. Reliability and validity of a new medication adherence rating scale (MARS) for the psychoses. Schizophr Res. (2000) 42:241–7. doi: 10.1016/S0920-9964(99)00130-9, PMID: 10785582

[24] Spitzer RL Kroenke K Williams JBW Löwe B. A brief measure for assessing generalized anxiety disorder: the GAD-7. Arch Intern Med. (2006) 166:1092–7. doi: 10.1001/archinte.166.10.1092, PMID: 16717171

[25] Riddell MA Edwards N Thompson SR Bernabe-Ortiz A Praveen D Johnson C. Developing consensus measures for global programs: lessons from the global Alliance for chronic diseases hypertension research program. Glob Health. (2017) 13:17. doi: 10.1186/s12992-017-0242-8, PMID: 28298233 PMC5353794

[26] Proctor E Silmere H Raghavan R Hovmand P Aarons G Bunger A. Outcomes for implementation research: conceptual distinctions, measurement challenges, and research agenda. Adm Policy Ment Health Ment Health Serv Res. (2011) 38:65–76. doi: 10.1007/s10488-010-0319-7, PMID: 20957426 PMC3068522

[27] Trapeznikova I. Measuring income inequality. IZA World Labor. (2019) 7:462. doi: 10.15185/izawol.462

[28] Schifano J Niederberger M. How Delphi studies in the health sciences find consensus: a scoping review. Syst Rev. (2025) 14:14. doi: 10.1186/s13643-024-02738-3, PMID: 39810238 PMC11734368

