# Supplementary material for: Standardized disease-related measures in diabetes research: results from a global consensus process
Source: Front Public Health. 2025 Jul 28;13:1580416. doi: 10.3389/fpubh.2025.1580416 (PMC12337128; doi:10.3389/fpubh.2025.1580416)
Supplement: Supplementary file 1 [file Table_1.docx]

***Supplementary material***

**S1 Table:** Descriptive statistics by domain. The 75^th^ percentile was used to determine which measures would be assessed further

| **Domain** | **Total** | **Mean** | **Median** | **IQR (25^th^ -75^th^)** |
| --- | --- | --- | --- | --- |
| 1. Demographics | 13 | 10 | 10 | 8-12 |
| 1. Anthropometry | 12 | 8 | 7 | 3-12 |
| 1. Behavioural measures | 12 | 6 | 4 | 3-9 |
| 1. Bio-chemical Measures | 12 | 3 | 2,5 | 1-5 |
| 1. Dietary Measures | 10 | 5 | 4 | 3-7 |
| 1. Health care utilisation | 10 | 3 | 3 | 2-4 |
| 1. Medical history | 13 | 5 | 4 | 1-11 |
| 1. Medication and adherence | 7 | 3 | 3 | 2-4 |
| 1. Physical activity | 12 | 10 | 9 | 8-11 |
| 1. Quality of life and stress | 8 | 2 | 2 | 1-3 |
| 1. Support systems | 3 | 1 | 1 | 0-1 |

IQR: Interquartile range
