# Supplementary material for: Standardized disease-related measures in diabetes research: results from a global consensus process
Source: Front Public Health. 2025 Jul 28;13:1580416. doi: 10.3389/fpubh.2025.1580416 (PMC12337128; doi:10.3389/fpubh.2025.1580416)
Supplement: Supplementary file 2 [file Table_2.docx]

***Supplementary material***

**S2 Table:** All measures assessed from Phase I and II and their final classification

|  | **Phase I: GACD Survey** | **Phase II: Online Modified Delphi Panel (ODP) Survey**  **Total # of experts = 32** | | | | **Final Recommendation** |
| --- | --- | --- | --- | --- | --- | --- |
| **Variable Name [GACD Data Dictionary Code]** | **n (%)** | **# Assessed Domain (%)** | **Core** | **Optional** | **Excluded** | **Classification** |
| **DOMAIN 1: Demographics** | **13 (100)** | **27 (84)** | **25 (93)** |  |  | **Core** |
| Participant ID Number [PIDNO] | 13 (100) |  | - | - | - | Core |
| Participant Age in years [DEM1] | 11 (85) |  | 22 (81) | 5 (19) |  | Core |
| Participant sex [DEM2] | 12 (92) |  | - | - | - | Core |
| Highest participant education [DEM3] | 12 (92) |  | - | - | - | Core |
| Participant Date of Birth [DEM1A] | 10 (77) |  | 9 (34) | 13 (50) | 4 (15) | Optional |
| Household size [DEM4] | 5 (38) |  |  | 15 (55) | 12 (45) | Optional |
| Household Income [DEM5a] | 7 (54) |  |  | 20 (74) | 7 (26) | Optional |
| Currently employed [OCCU1] | 10 (77) |  | 17 (63) | 10 (37) |  | Optional |
| Occupation [OCCU2] | 9 (69) |  |  | 18 (67) | 9 (33) | Optional |
| Participant age estimated [DEM1B] | 4 (31) |  |  | 10 (37) | 17 (63) | Excluded |
| **DOMAIN 2: Anthropometry** | **12 (92)** | **23 (72)** | **22 (96)** |  |  | **Core** |
| Systolic blood pressure [CLIN3] | 12 (100) |  | - | - | - | Core |
| Diastolic blood pressure [CLIN4] | 12 (100) |  | - | - | - | Core |
| Participant height [CLIN7] | 12 (100) |  | - | - | - | Core |
| Participant waist circumstance [CLIN11] | 11 (92) |  | - | - | - | Core |
| Participant weight [CLIN9] | 12 (100) |  | - | - | - | Core |
| Cuff size [CLIN2] | 7 (58) |  | 12 (54) | 9 (41) | 1 (5) | Optional |
| Pulse rate [CLIN5] | 7 (58) |  | 6 (27) | 13 (59) | 3 (14) | Optional |
| Participant hip circumference [CLIN13] | 6 (50) |  |  | 13 (56) | 10 (44) | Optional |
| **DOMAIN 3: Behavioural Measures** | **12 (92)** | **25 (78)** | **25(100)** |  |  | **Core** |
| Currently smoke cigarettes [SMK1] | 12 (100) |  | - | - | - | Core |
| Currently use smokeless tobacco / chewing tobacco/ snuff [SMK2] | 7 (58) |  | 20 (80) | 4 (16) | 1 (4) | Core |
| Frequency of tobacco (smoking or smokeless) use [SMK3] | 10 (83) |  | - | - | - | Core |
| Alcohol frequency over the past 12 months [ALC3] | 9 (75) |  | - | - | - | Core |
| Blood sugar healthcare professional [BSL2]* | 4 (33) |  | 22 (88) | 2 (8) | 1 (4) | Core |
| Smoking/chewing tobacco use over 100 times in a lifetime [SMK4] | 1 (8) |  |  | 14 (56) | 11 (44) | Optional |
| Exposure to passive smoke [SMK5] | 3 (25) |  |  | 14 (56) | 11 (44) | Optional |
| EVER consumed any alcohol (beer, wine, spirits) [ALC1] | 7 (58) |  | 13 (52) | 8 (32) | 4(16) | Optional |
| Consumed any alcohol (beer, wine, spirits) within the past 12 months [ALC2] | 8 (67) |  | 15 (60) | 6 (24) | 4(16) | Optional |
| Alcohol cessation [ALC4] | 0 (0) |  |  | 14 (56) | 11 (44) | Optional |
| Foot exam HC professional [FTEX1]* | 4 (33) |  | 11 (44) | 12 (48) | 2 (8) | Optional |
| Foot exam self [FTEX2]* | 4 (33) |  | 11 (44) | 7 (28) | 7 (28) | Optional |
| Blood sugar self [BGL1]* | 2 (17) |  |  | 17 (68) | 8 (32) | Optional |
| Eye exam professional [OPTH1] | 3 (25) |  |  | 16 (64) | 9 (36) | Optional |
| **DOMAIN 4: Biochemical Measures** | **12 (92)** | **23 (72)** | **19 (83)** |  |  | **Core** |
| Fasting blood glucose measure in the past 12 hours [BGL1] | 5 (42) |  | - | - | - | Core |
| Unit for fasting blood glucose measure [BGL4] | 7 (58) |  | - | - | - | Core |
| Baseline time for blood glucose concentration [OGTT1] | 4 (33) |  | 20 (90) | 1 (5) | 1 (5) | Core |
| Unit of baseline glucose concentration [OGTT2] | 5 (42) |  | - | - | - | Core |
| Glycated hemoglobin concentration [HBA1C] | 9 (75) |  | - | - | - | Core |
| Total cholesterol [CHOL1] | 5 (42) |  | - | - | - | Core |
| High-density lipoprotein (HDL) [CHOL2] | 5 (42) |  | - | - | - | Core |
| Cholesterol: High-density lipoprotein ratio [CHOL4] | 5 (42) |  | - | - | - | Core |
| Unit for 2hr glucose concentration [OGTT7] | 3 (25) |  | 8 (36) | 13 (59) | 1 (5) | Optional |
| Albumin: Creatinine ratio (urine spot collection) [ACR1] | 2 (17) |  |  | 14 (61) | 9 (39) | Optional |
| Low-density lipoprotein (LDL) [CHOL3] | 4 (33) |  | 13 (59) | 7 (32) | 2 (9) | Optional |
| Time of day fasting blood glucose measure was taken [BGL3] | 2 (17) |  |  | 10 (43) | 14 (57) | Excluded |
| Taken medicine before fasting blood glucose measure [BGL5] | 1 (8) |  |  | 1 (4) | 22 (96) | Excluded |
| 24hr urine volume [URN1] | 0 (0) |  |  | 4 (17) | 19 (83) | Excluded |
| Completeness of the 24hr urine collection [URN2] | 0 (0) |  |  | 0 (0) | 0 (0) | Excluded |
| Na concentration [URN3] | 0 (0) |  |  | 0 (0) | 0 (0) | Excluded |
| Total oral glucose load [OGTT3] | 1 (8) |  |  | 2 (9) | 21 (91) | Excluded |
| Time of 1hr blood draw [OGTT4] | 1 (8) |  |  | 4 (17) | 19 (83) | Excluded |
| Unit for 1hr glucose concentration [OGTT5] | 2 (17) |  |  | 4 (17) | 19 (83) | Excluded |
| Time of 2hr blood draw [OGTT6] | 1 (8) |  |  | 6 (26) | 17 (74) | Excluded |
| **DOMAIN 5: Dietary measures** | **10 (77)** | **21 (66)** | **20 (95)** |  |  | **Core** |
| Frequency of fruit consumption in a week [DIET6] | 9 (90) |  | - | - | - | Core |
| Frequency of fruit servings on a day when fruit is consumed [DIET7] | 7 (70) |  | - | - | - | Core |
| Frequency of vegetable consumption in a week [DIET8] | 9 (90) |  | - | - | - | Core |
| Frequency of vegetable services on a day when vegetables are consumed [DIET9] | 7 (70) |  | - | - | - | Core |
| Added salt at the table [DIET1] | 5 (50) |  | 11 (52) | 7 (33) | 3 (14) | Optional |
| Added salt during cooking [DIET2] | 3 (30) |  |  | 11 (52) | 10 (48) | Optional |
| Frequency of fried vegetables in a week [DIET10] | 2 (20) |  |  | 14 (67) | 7 (33) | Optional |
| Frequency of protein consumption in a week [DIET11] | 4 (40) |  | 7 (33) | 12 (57) | 2 (10) | Optional |
| Frequency of fish consumption in a week [DIET12] | 3 (30) |  |  | 12 (57) | 9 (43) | Optional |
| Frequency of nuts, legumes or seeds in a week [DIET13] | 5 (50) |  | 8 (38) | 12 (57) | 1 (5) | Optional |
| Frequency of dairy consumption in a week [DIET14] | 3 (30) |  |  | 13 (62) | 8 (38) | Optional |
| Frequency of deep-fried food consumption in a week [DIET15] | 4 (40) |  | 11 (52) | 10 (48) |  | Optional |
| Oily used for food preparation [DIET16] | 5 (50) |  | 10 (48) | 9 (43) | 2 (9) | Optional |
| Frequency of salty food consumption [DIET3] | 2 (20) |  |  | 6 (29) | 15 (71) | Excluded |
| Self-estimation of salt consumption [DIET4] | 1 (10) |  |  | 4 (19) | 17 (81) | Excluded |
| Frequency of teaspoons of added salt [DIET5] | 1 (10) |  |  | 5 (24) | 16 (76) | Excluded |
| **DOMAIN 6: Health Care Utilisation** | **10 (77)** | **27 (84)** | **22 (96)*** |  |  | **Core** |
| Medical advice in the last 3 months [HCU1a] | 5 (50) |  | - | - | - | Core |
| Frequency of medical advice in the last 3 months [HCU1b] | 4 (40) |  | - | - | - | Core |
| Admitted to the hospital in the last year [HCU3a] | 4 (40) |  | - | - | - | Core |
| Health insurance [HCU8a] | 5 (50) |  | - | - | - | Core |
| Type of health insurance [HCU8b] | 5 (50) |  | - | - | - | Core |
| Payment of health insurance [HCU8c] | 4 (40) |  | - | - | - | Core |
| Treatment location [HCU1d] | 3 (30) |  | 13 (50) | 12 (46) | 1 (4) | Optional |
| Who provided treatment [HCU1e] | 3 (30) |  | 11 (44) | 10 (40) | 4 (16) | Optional |
| Treatment payment [HCU1f] | 3 (30) |  | 9 (36) | 14 (56) | 2 (8) | Optional |
| Last routine check-up [HCU4a] | 3 (30) |  | 11 (44) | 10 (40) | 4 (16) | Optional |
| Frequency of medical advice in the last 4 weeks [HCU1c] | 2 (20) |  |  | 8 (30) | 19 (70) | Excluded |
| Pattern of health seeking behavior [HCU2a] | 0 (0) |  |  | 6 (22) | 21 (78) | Excluded |
| Differences in health seeking behavior patterns [HCU2b] | 0 (0) |  |  | 4 (15) | 23 (85) | Excluded |
| Type of facility for in-patient care [HCU3b] | 2 (20) |  |  | 6 (22) | 21 (78) | Excluded |
| Length of in-patient stay [HCU3c] | 2 (20) |  |  | 6 (22) | 21 (78) | Excluded |
| Payment for in-patient stay [HCU3d] | 2 (20) |  |  | 7 (26) | 20 (74) | Excluded |
| Reason for no routine check-ups [HCU4b] | 1 (10) |  |  | 4 (15) | 23 (85) | Excluded |
| Ease of transport to the health facility [HCU5] | 0 (0) |  |  | 6 (22) | 21 (78) | Excluded |
| Type of transport to the health facility [HCU6] | 1 (10) |  |  | 6 (22) | 21 (78) | Excluded |
| Time to health facility [HCU7] | 2 (20) |  |  | 5 (19) | 22 (81) | Excluded |
| **DOMAIN 7: Medical History** | **13 (100)** | **24 (75)** | **22 (96)** |  |  | **Core** |
| History of hypertension [MED1] | 12 (92) |  | - | - | - | Core |
| Hypertension medication [MED2] | 11 (85) |  | - | - | - | Core |
| History of CVD: doctor informed [MED4a] | 8 (62) |  | 20 (83) | 3 (13) | 1 (4) | Core |
| History of stroke: doctor informed [MED5] | 7 (54) |  | 21 (88) | 3 (12) |  | Core |
| History of diabetes: doctor informed [MED3] | 11 (85) |  | - | - | - | Core |
| Diabetes medication [MED8] | 12 (92) |  | - | - | - | Core |
| Insulin use [MED9] | 8 (62) |  | 20 (83) | 4 (17) |  | Core |
| History of chronic kidney disease: doctor informed [MED7] | 6 (46) |  | 20 (83) | 4 (17) |  | Core |
| Foot ulcer in past year [FU01] | 4 (31) |  | 13 (57) | 9 (39) | 1 (4) | Optional |
| Coronary artery bypass surgery [MED4b] | 3 (23) |  |  | 11 (46) | 13 (54) | Excluded |
| Coronary angioplasty or a stent inserted [MED4c] | 3 (23) |  |  | 9 (38) | 15 (62) | Excluded |
| Definition of hypertension [KNO1] | 1 (8) |  |  | 9 (38) | 15 (62) | Excluded |
| Dangers of hypertension [KNO2] | 1 (8) |  |  | 5 (21) | 19 (79) | Excluded |
| Impact of lowering blood pressure [KNO3] | 0 (0) |  |  | 6 (25) | 18 (75) | Excluded |
| Awareness of blood pressure [KNO4] | 1 (8) |  |  | 6 (25) | 18 (75) | Excluded |
| Awareness of systolic blood pressure [KNO5] | 1 (8) |  |  | 7 (29) | 17 (71) | Excluded |
| Awareness of diastolic blood pressure [KNO6] | 1 (8) |  |  | 7 (29) | 17 (71) | Excluded |
| Date of foot ulcer [FU02] | 0 (0) |  |  | 8 (33) | 16 (67) | Excluded |
| **DOMAIN 8: Medication and Adherence** | **7 (100)** | **24 (75)** | **19 (90)** |  |  | **Core** |
| Forget to take medication 1 [ADHR1]* | 5 (71) |  | - | - | - | Core |
| Adherence to medication when feeling good [ADHR4]* | 4 (57) |  | - | - | - | Core |
| Clinical data collection month & year [CLI_MON] & [CLI_YR] | 4 (57) |  | - | - | - | Core |
| Carelessness to taking medication [ADHR2]* | 3 (43) |  | 11 (46) | 6 (25) | 7 (29) | Optional |
| Side effects influence medication adherence [ADHR3]* | 3 (43) |  | 12 (50) | 7 (29) | 5 (21) | Optional |
| Injectables (insulin) [CLI1A-1] | 3 (43) |  | 14 (61) | 5 (22) | 4 (17) | Optional |
| Start date of injectables [CLI1A-2] | 3 (43) |  | 9 (41) | 10 (45) | 3 (14) | Optional |
| Oral hypoglycemic or antihyperglycemic agents [CLI2-1] | 3 (43) |  | 13 (59) | 6 (27) | 3 (14) | Optional |
| Type of oral hypoglycemic or antihyperglycemic agents and dose [CLI2-2] | 4 (57) |  | 11 (48) | 9 (29) | 3 (13) | Optional |
| Traditional or complementary medicines [CLI3-1] | 3 (43) |  | 8 (35) | 11 (48) | 4 (17) | Optional |
| Injectables other than insulin [CL1B-1] | 2 (29) |  |  | 5 (21) | 19 (79) | Excluded |
| Type of drug if injectables other than insulin [CL1B-2] | 2 (29) |  |  | 2 (8) | 22 (92) | Excluded |
| Start date of injectables other than insulin [CL1B-3]** | 3 (43) |  | 10 (43) | 6 (26) | 7 (31) | Excluded |
| Type of traditional or complementary medicines and dose [CLI3-2] | 2 (29) |  |  | 3 (13) | 21 (87) | Excluded |
| **DOMAIN 9: Physical Activity** | **12 (100)** | **23 (72)** | **20 (100)** |  |  | **Core** |
| Physical activity time [PHY1] | 10 (83) |  | 22 (100) | - |  | Core |
| Walking/cycling [PHY2] | 8 (67) |  |  | 12 (52) | 11 (48) | Optional |
| Sedentary behaviour [PHY16] | 8 (67) |  |  | 11 (48) | 12 (52) | Excluded |
| **DOMAIN 10: Quality of Life and Stress** | **8 (100)** | **21 (66)** | **19 (95)** |  |  | **Core** |
| General perception [QOL1] | 6 (75) |  | - | - | - | Core |
| Health assessment [QOL2] | 7 (88) |  | - | - | - | Core |
| Nervous [QOL11a]* | 4 (50) |  | - | - | - | Core |
| Worry [QOL11c]* | 3 (38) |  | - | - | - | Core |
| Difficulties [QOL11h] | 4 (50) |  | - | - | - | Core |
| Stress [QOL8b] | 2 (25) |  | 10 (48) | 10 (48) | 1 (4) | Optional |
| Ongoing difficulties work [QOL5a] | 2 (25) |  | 7 (35) | 12 (60) | 1 (5) | Optional |
| Constant worry [QOL11b]* | 2 (25) |  | 7 (33) | 12 (57) | 2 (10) | Optional |
| Trouble relaxing [QOL11d]* | 2 (25) |  | 7 (33) | 12 (57) | 2 (10) | Optional |
| Restless [QOL11e]* | 2 (25) |  | 4 (19) | 15 (71) | 2 (10) | Optional |
| Annoyed [QOL11f]* | 2 (25) |  | 5 (24) | 14 (67) | 2 (9) | Optional |
| Ongoing problems [QOL3a] | 1 (12) |  |  | 8 (38) | 13 (62) | Excluded |
| Ongoing problems - time [QOL3b] | 1 (12) |  |  | 8 (38) | 13 (62) | Excluded |
| Ongoing problems finance [QOL6a] | 1 (12) |  |  | 10 (48) | 11 (52) | Excluded |
| Ongoing problems finance - time [QOL6b] | 1 (12) |  |  | 9 (43) | 12 (57) | Excluded |
| Ongoing difficulties relationship [QOL7a] | 1 (12) |  |  | 10 (48) | 11 (52) | Excluded |
| Ongoing difficulties relationship - time [QOL7b] | 1 (12) |  |  | 8 (38) | 13 (62) | Excluded |
| Ongoing problems degree [QOL8a] | 1 (12) |  |  | 6 (29) | 15 (71) | Excluded |
| Ongoing difficulties work - time [QOL5b] | 1 (12) |  |  | 8 (38) | 13 (62) | Excluded |
| Afraid [QOL11g]* | 1 (12) |  |  | 6 (29) | 15 (71) | Excluded |
| **DOMAIN 11: Support Systems** | **3 (100)** | **18 (56)** | **14 (82)** |  |  | **Optional** |
| Support friends [QOL9a] | 1 (33) |  | 10 (56) | 7 (39) | 1 (5) | Optional |
| Support spouse [QOL9b] | 1 (33) |  | 13 (72) | 5 (28) |  | Optional |
| Support parent [QOL9c] | 1 (33) |  | 6 (33) | 12 (67) |  | Optional |
| Support sibling [QOL9d] | 1 (33) |  | 4 (24) | 13 (76) |  | Optional |
| Support relative [QOL9e] | 1 (33) |  | 5 (29) | 10 (59) | 2 (12) | Optional |
| Financial Support friend [QOL10a] | 0 (0) |  |  | 12 (67) | 6 (33) | Optional |
| Financial Support spouse [QOL10b] | 0 (0) |  |  | 11 (61) | 7 (39) | Optional |
| Financial Support parent [QOL10c] | 0 (0) |  |  | 10 (56) | 8 (44) | Optional |
| Financial Support sibling [QOL10d] | 0 (0) |  |  | 9 (50) | 9 (50) | Optional |
| Financial Support relative [QOL10e] | 0 (0) |  |  | 11 (61) | 7 (39) | Optional |

*indicates it is part of a validated scale. In Phase I (GACD Survey), the n refers to the number of projects collecting that variable. In Phase II, the n refers to the number of experts who responded to that variable.
